# Twiddler’s syndrome in spinal cord stimulation

**DOI:** 10.1007/s00701-015-2627-x

**Published:** 2015-11-17

**Authors:** Rafid Al-Mahfoudh, Yuen Chan, Hsu Pheen Chong, Jibril Osman Farah

**Affiliations:** The Walton Centre for Neurology & Neurosurgery, Lower Lane, Liverpool, L9 7LJ UK; South East Neurosurgery and Spinal Surgery, Brighton and Sussex University Hospitals NHS Trust, Brighton, UK

**Keywords:** Twidder’s syndrome, Spinal cord stimulation, IPG, Failed back surgery syndrome

## Abstract

**Background:**

The aims are to present a case series of Twiddler’s syndrome in spinal cord stimulators with analysis of the possible mechanism of this syndrome and discuss how this phenomenon can be prevented.

**Method:**

Data were collected retrospectively between 2007 and 2013 for all patients presenting with failure of spinal cord stimulators. The diagnostic criterion for Twiddler’s syndrome is radiological evidence of twisting of wires in the presence of failure of spinal cord stimulation.

**Results:**

Our unit implants on average 110 spinal cord stimulators a year. Over the 5-year study period, all consecutive cases of spinal cord stimulation failure were studied. Three patients with Twiddler’s syndrome were identified. Presentation ranged from 4 to 228 weeks after implantation. Imaging revealed repeated rotations and twisting of the wires of the spinal cord stimulators leading to hardware failure.

**Conclusions:**

To the best of our knowledge this is the first reported series of Twiddler’s syndrome with implantable pulse generators (IPGs) for spinal cord stimulation. Hardware failure is not uncommon in spinal cord stimulation. Awareness and identification of Twiddler’s syndrome may help prevent its occurrence and further revisions. This may be achieved by implanting the IPG in the lumbar region subcutaneously above the belt line. Psychological intervention may have a preventative role for those who are deemed at high risk of Twiddler’s syndrome from initial psychological screening.

## Introduction

Twiddler’s syndrome is rare clinical condition. It is commonly been reported in cardiac pacemakers and implantable cardioverter defribrillators [5, 7, 12, 13, 19, 24, 27, 28, 30, 32, 39]. In neurosurgical practice, Twiddler’s syndrome has been reported in deep brain stimulation but never in spinal cord stimulation (SCS) [3, 9, 16, 17, 21, 31, 34]. To the best of our knowledge, we present the first reported case series of hardware malfunction due to Twiddler’s syndrome in SCS. Bayliss et al. [5] were the first to report this phenomenon in cardiac pacemakers in 1968. In their case report, it was found that the rotation of the wires was a result of twiddling by the patient. Twiddler’s syndrome can be a conscious or subconscious manipulation of an implantable pulse generator (IPG) within its subcutaneous pocket. This ultimately leads to hardware failure, which is often the mode of presentation. There are many components of an SCS and each providing a possible point of failure. Hardware failure can be due to breakage, infection, migration [36]. In Twiddler’s syndrome in addition to the radiological evidence, there is raised impedance leading to system failure, loss of capture and a recurrence of symptoms.

## Materials and methods

An average of 110 spinal cord stimulators are inserted per year in our unit. All patients presenting with failure of spinal cord stimulators between 2007 and 2013 were reviewed to identify those with evidence of Twiddler’s syndrome. Three patients with radiological evidence of Twiddler’s syndrome were identified. Informed consent was obtained from these three patients to be included in this study. Radiological evidence consisted of twisting and rotation of the wires. Their case notes, images and management were reviewed. All patients underwent a pain management program (PMP) and psychological assessment prior to considering implanting an SCS. Our department’s policy is to obtain routine postoperative radiological images for all cases of SCS as a baseline. In cases of clinical failure of the device, investigations include interrogating the device and radiological imaging to exclude disconnections, lead breaks and other causes of hardware failure.

## Results

### Case report 1

A 54-year-old woman with failed back surgery syndrome following a lumbar microdiscectomy had a spinal cord stimulator inserted in October 2009. A Specify 5-6-5 electrode (Medtronic, Minneapolis, MN, USA) was implanted with the connectors tunnelled subcutaneously and connected to the IPG in the right iliac fossa. There were no intraoperative or postoperative complications. The patient experienced good relief of lower limb pain with stimulation.

At the point of insertion, she weighed 78.8 kg, with a height of 157 cm (body mass index [BMI], 31.9). Her pre-PMP assessment revealed moderate levels of depression and high levels of pain-related disability. Her Beck Depression Inventory score was 18 (mild). She had a Pain Anxiety Symptoms Scale of 60 (mean, 94; SD, 39; range 0–200, where 0 = no pain anxiety and 200 = severe pain anxiety).

**Fig. 1:**
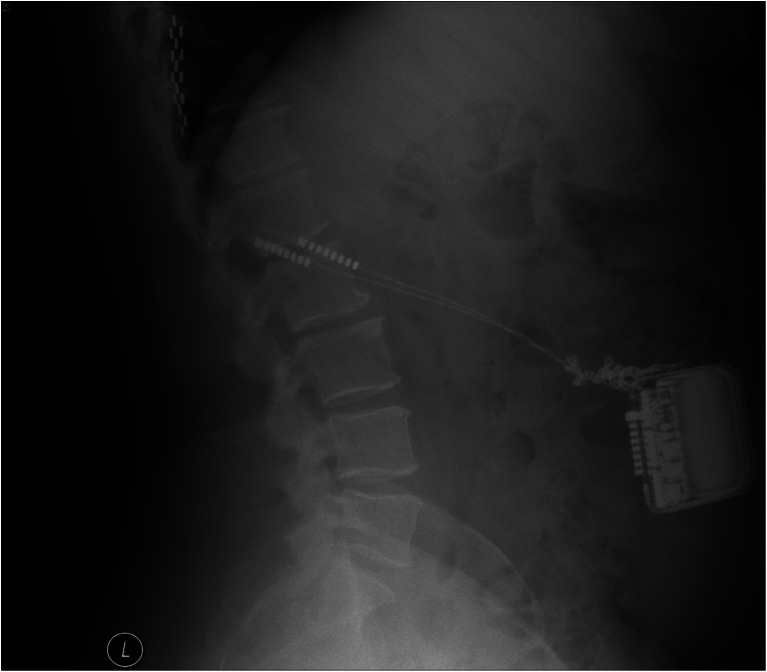
Lateral plain abdominal X-ray showing twisting of the connecting wires adjacent to the IPG

In October 2011, she underwent reprogramming with failure to achieve satisfactory stimulation. Her imaging revealed twisting of the connecting wires adjacent to the pulse generator (Fig. 1). As it was still partially functioning, she chose to defer revision surgery. Eventually the device failed completely. In May 2012, her IPG was repositioned with new connector leads. The connector wires were found knotted and twisted intraoperatively. The new wires were tunnelled to the right lumbar region above the belt line. No further problems have been reported on follow-up.

### Case report 2

A 31-year-old woman with failed back surgery syndrome following a right lumbar microdiscectomy. Her weight was 93.1 kg and height 165 cm (BMI, 34.2). In her pre-PMP assessment, she had moderate depression, moderate pain-related disability and average scores on pain distress and intensity. A percutaneous trial of spinal cord stimulation was judged positive and therefore the patient opted for permanent spinal cord stimulator insertion. An eight-electrode surgical lead was implanted with the IPG in the iliac fossa. Postoperatively there was good reduction in pain scores, analgesic usage and mobility; plain X-ray indicated satisfactory electrode placement.

**Fig. 2:**
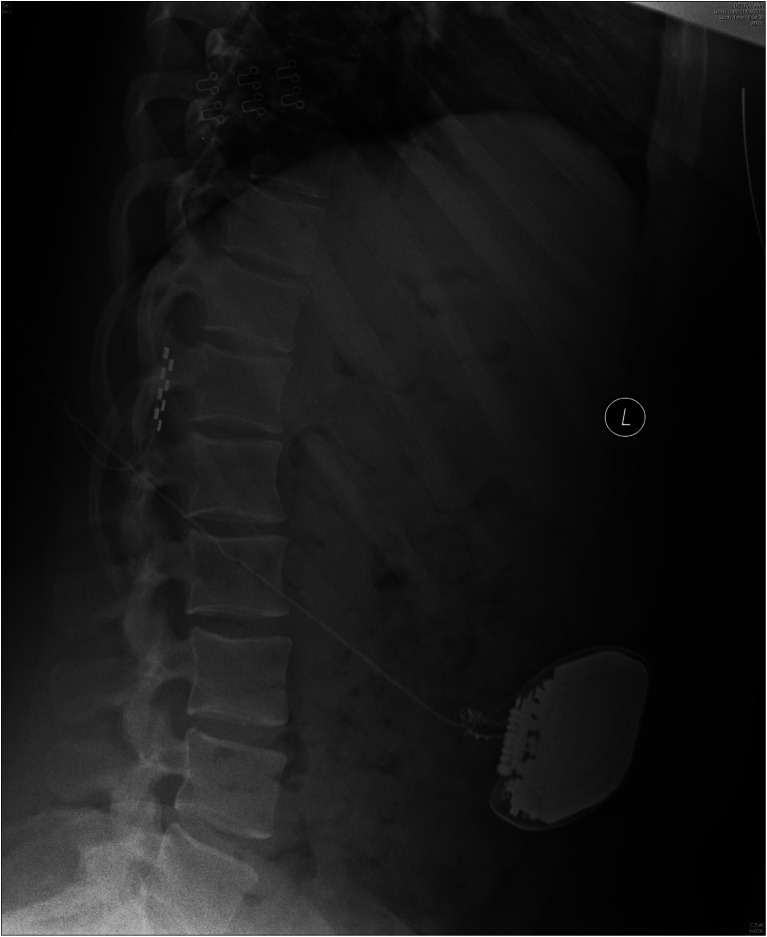
Lateral plain abdominal X-ray showing multiple twists of the connecting wires adjacent to the IPG

Four weeks after insertion, symptoms recurred to preoperative levels. Interrogation of the system revealed high impedances suggestive of electrode failure. X-ray of the system indicated an abnormal twisting of the wiring between the implanted abdominal pulse generator and the spinal electrode. The subcutaneous pulse generator had undergone repeated rotation, twisting the connecting leads to the point of fracture (Fig. 2).

**Fig. 3:**
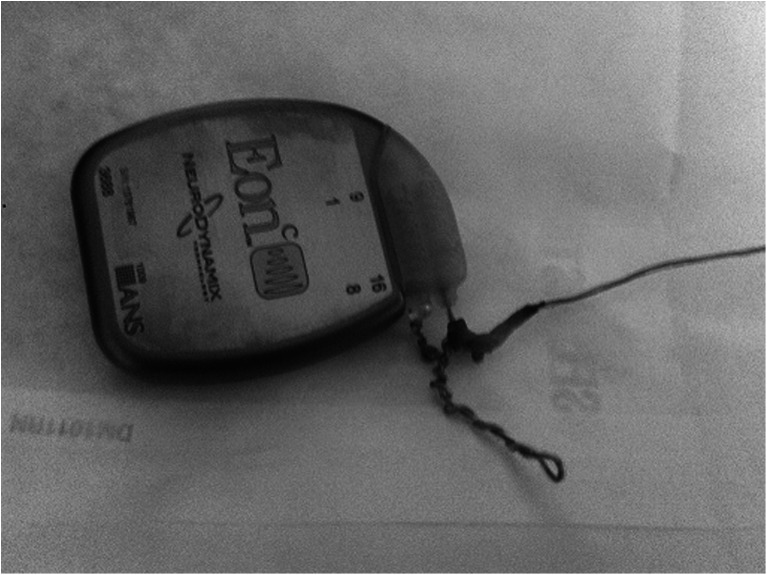
IPG with twisted wires after removal from patient

Surgical revision of the system was performed. The X-ray findings were confirmed intraoperatively. The IPG had rotated on its axis many times (Fig. 3). A new pulse generator was implanted in her right lumbar region above the iliac crest and belt line. Postoperatively, the patient reported restoration of good pain relief.

### Case 3

A 50-year-old woman who initially presented with cauda equina syndrome secondary to a L5/S1 disc prolapse was later diagnosed with failed back surgery syndrome. She weighed 96.95 kg, with a height of 1.57 (BMI, 39.3). This lady had a pain anxiety and symptom scale score of 152 (mean, 94; SD, 39; range, 0–200). On the Becks depression scale, she scored 48 (severe depression).

She had a trial of spinal cord stimulation in 2008, which achieved good control of her pain symptoms. She proceeded to have a spinal cord stimulator (5-6-5 electrode) inserted permanently at the level of T10-12 with connectors tunnelled to the right iliac fossa.

She had good control of her back pain initially; however, during follow-up her pain control deteriorated. In late 2009, she complained of her IPG being mobile and catching on her clothes and it was re-sited at that point. In 2013, she presented with SCS failure. X-rays showed signs of Twiddler’s syndrome. On revision of her SCS, the whole extension wires were badly twisted and damaged. She had a replacement extension set tunnelled and connected to the main electrodes, with the IPG repositioned to the lumbar region.

**Fig. 4:**
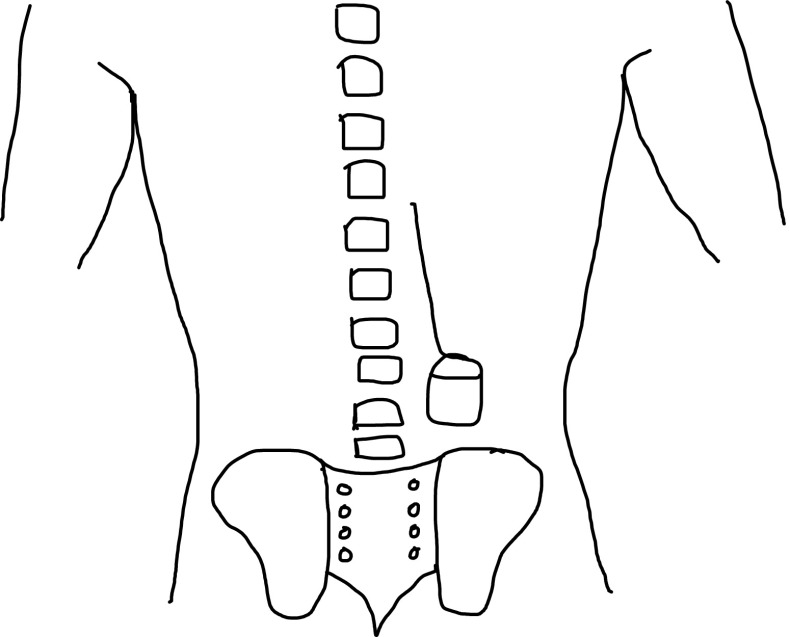
Schematic diagram illustrating IPG site for revision cases

In all three cases the surgical technique included anchoring the lead with the included manufacturer anchoring system. This was by suturing the anchoring system to the lumbar fascia. It was not our policy to anchor the pulse generator in its subcutaneous pocket in the primary surgical procedure. All three patients denied manipulating the IPG. In the revision procedure in all three cases, the IPG was implanted in the lumbar region (Fig. 4).

## Discussion

To the best of our knowledge and following a literature research by two independent investigators, we present the first reported case series on Twiddler’s syndrome in SCS. All patients were female, aged between 31 and 54 years and all patients had varying degrees of depression and anxiety. Their BMI ranged between 31.9 and 39.3, which falls into the obese category according to the WHO classification [37]. There was no pattern to the timing of presentation, which ranged from 4 to 228 weeks after implantation. Our patients underwent validated psychological testing, which consisted of the Becks Depression Inventory [6] and the Pain Anxiety Symptoms Scale [29] prior to treatment which revealed varying degrees of depression and fear of pain.

The first SCS was implanted by Shealy et al. in 1967 [38]. Hardware failure is not an uncommon complication with SCS and it is reported to occur most commonly within the first 2 years after implantation [25, 43]. In Twiddler’s syndrome with other implantable devices, presentation is reported between 6 months and 3 years [3, 9, 16, 17, 21, 31, 34, 39].

Hardware-related complications in SCS have different aetiologies, including lead migration/fracture, electrical shorting-out with cessation of stimulation, infection, decreased stimulation and battery end of life. Such complications have been reported to be between 22 and 27.2 % is SCS [25, 41]. Hardware malfunction in Twiddler’s syndrome is due to lead coiling, which can cause displacement and lead fracture [9, 16, 20, 34]. Twisting of the wires in such a manner has not been reported with SCS previously [8, 23, 26].

Twiddler’s syndrome typically presents with loss of stimulation, resulting in a recurrence of symptoms [32, 34]. There may be pain along the course of the wires due to movement and traction of the wires. Although Twiddler’s syndrome is a rare condition, there may be an element of under-reporting of Twiddler’s syndrome due to the lack of awareness of this problem in SCS.

Twiddler’s syndrome has most commonly been described with cardiac pacemakers and defibrillators [5, 7, 12, 13, 19, 24, 27, 28, 30, 32, 39]. More recently it has been described in deep brain stimulation [3, 9, 16, 17, 21, 31, 34]. Table 1 summarises all cases of Twiddler’s syndrome reported in the literature with different implants. The aetiology of Twiddler’s syndrome is not completely understood; however, some have found an association with obese women [7, 12, 16, 24, 34]. With increased subcutaneous tissue, rotation of the device may occur during normal activity, which in turn can lead to wire coiling on the IPG. In one case series [17], the patients admitted to actively twisting the IPG. However, in most reports patients deny manipulating the IPG. Physical activity has been postulated as a causative factor in IPGs implanted in the pectoral region [7, 16, 34]. An association has been noted with neurological and psychological conditions such as dementia, obsessive-compulsive disorders, seizures, depression and anxiety [9, 10, 20, 22, 34]. All patients in this series denied manipulating their IPGs. All our patients with Twiddler’s had moderate to severe depression on preoperative psychological screening.

**Table 1:** Reported cases of Twiddler’s syndrome in the literature

Author/Year	Country	Device involved	Baseline characteristics	Causes	Intervention
Frizell et al. 2015 [14]	USA	Pacemaker/ defibrillator	53-year-old female	Not stated but patient was frequently touching her incision Denied manipulation	Lead revision
Dattilo et al. 2015 [11]	Italy	ICD	76-year-old male	Manipulation by patient with presence of scratches around pockets	Tied device with silk to pectoral muscle, repair sleeves to leads
Silva et al. 2014 [40]	Portugal	DBS – bilateral subthalamic nucleus stimulation	65-year-old female Parkinson’s disease Mild medically compensated reactive depression	Denied manipulation No causative factor identified	Revision surgery to replace damaged leads
Raissuni et al. 2014 [35]	France	ICD	70-year-old female	Denied manipulation No causative factor identified	Device replaced and sutured tightly to underlying muscle
Garweg et al. 2014 [15]	Belgium	Dual chamber cardioverter defibrillation	72-year-old male	Spontaneous Denied manipulation	Generator fixed in pocket with ligature
Stryjewski et al. 2014 [42]	Poland	Pacemaker	77-year-old male Psycho-organic syndrome	Not stated	New lead inserted and pacemaker fixed to muscle with non-absorbable suture
Bali et al. 2013 [4]	India	Pacemaker	Elderly female	Over enthusiastic masseuse	Repositioning leads with standard suturing technique
Trout et al. 2013 [44]	USA	Vagal nerve stimulator	8-year-old male ADHD Lifelong intractable seizures Autistic behaviours Global developmental arrest	Manipulation of the device by child	Not stated
Liang et al. 2013 [28]	USA	Pacemaker	52-year-old female	Not stated	Lead revision and device reinforcement in its pocket to fascia or subpectoral placement
Meghetti et al. 2013 [31]	Italy	DBS – posteroventral globus pallidus	43-year-old female	Manipulation of IPG by patient	IPG positioned in a submuscular pocket and fixed to fascia
Ahmed et al. 2013 [1]	UK	ICD	60-year-old male	Not stated	Repositioning of leads and anchoring the device to the pectoral muscle
Gonzalez Bermudez et al. 2012 [18]	Spain	ICD	74-year-old female	Denied manipulation. Spontaneous rotation of device	Repositioning of leads and generator attached to pectoral muscle
Grapsa et al. 2013 [19]	UK	Pacemaker	86-year-old male Parkinson’s syndrome	Patient manipulated pacing box due to Parkinson’s disease	Reprogramming of the device
Ali et al. 2012 [2]	USA	ICD	44-year-old female Obesity	Used left arm to carry heavy shopping bags (implantation of ICD in left pectoral region) Occasionally touched and scratched skin Denied manipulation	Lead uncoiled and repositioned. IPG repositioned.
Penn et al. 2012 [34]	USA	DBS – bilateral anterior nuclei of the thalamus	21-year-old female Epilepsy, no psychiatric history	Not stated	New extensions inserted and sutured. Fascial suture to anchor IPG in a polyester pouch
Pavlidis et al. 2011 [33]	Greece	Pacemaker	82-year-old female Severely impaired mental status	Conscious or unconscious manipulation of the pulse generator	Not stated
Astradsson et al. 2010 [3]	UK	DBS	65-year-old female	Denied manipulation Loose IPG in a large pocket with conscious or unconscious twiddling	Revision of leads secured underneath the IPG and fixed. Further revision and IPG secured with a prolene mesh
Bayliss et al. 1968 [5]	Canada	Pacemaker	79-year-old female	Manipulation of device Loose pocket	Repositioning of the leads
Bracke et al. 2005 [7]	Netherlands	ICD	60/59/50-year-old patients	Denied manipulation All had history of recent strenuous physical exercise, one recurred after swimming No obesity	Repositioning of leads
Burdick et al. 2010 [9]	USA	DBS	79-year-old female with depression with episodes of suicidal ideation, anxiety 74-year-old male, BMI 46.7 71-year-old female, Parkinson’s disease	Denies manipulation. Patient felt spontaneous movement of IPG	Leads and IPG anchored the fascia with silk sutures
Castillo et al. 2006 [10]	USA	Pacemaker	76-year-old male Dementia	Manipulation of the device	Conversion of pacemaker mode and restrictive recommendations to prevent manipulation
De Buitleir et al. 1996 [12]	USA	ICD	54-year-old female Obesity	Denies manipulation but on waking in the morning notices the ICD sitting on the side	Lead revision
Femenia et al. 2010 [13]	Argentina	Pacemaker	74-year-old female	Moving arms energetically	IPG anchored to pocket
Geissinger et al. 2007 [16]	USA	DBS	65-year-old female	Patient felt IPG shifted	IPG placed into a polyester pouch and secure to surrounding tissue
Gelabert-Gonzalez et al. 2010 [17]	Spain	DBS	68-year-old female, Parkinson’s disease 65-year-old female	Patient admitted twisting the generator.	Replacement of the electrode and extension wires
Harel et al. 2008 [20]	Israel	Pacemaker	69-year-old female	Not stated	Re-implantation of electrode
Israel et al. 2008 [21]	Israel	DBS – bilateral thalamic surgery	65-year-old female No psychiatric history	Patient complained of an itching sensation over incision and was advised to gently massage the incision	Leads revision and IPG sutured to periosteum and fascia with silk sutures, subcutaneous pocket contracted by sutures
Jaafari et al. 2009 [22]	France	ICD	47-year-old male Obsessional personality traits	Since the implantation the patient had increased anxiety and described fears of having chest pain and of dying as well as receiving shocks which led to compulsive checking behaviour of ICD	Patient underwent cognitive behavioural therapy and antidepressants
Lal et al. 1990 [27]	USA	Pacemaker	89-year-old male	Not stated	Repositioned leads and re-anchored using suture sleeve and pacemaker suture to pectoralis minor muscle
Mehta et al. 1992 [30]	USA	ICD	45-year-old, sex not stated Obese	Denied manipulation but reported local discomfort and excessive device mobility	Replacement of leads and generator re-implanted with a Dacron patch
Nicholson et al. 2003 [32]	USA	Pacemaker	75-year-old male	Patient had been spinning the pulse generator in its surgical pocket	Pacing leads replaced and additional suture were added to secure the pacemaker body to the fascia in the surgical pocket
Sidhu et al. 2009 [39]	USA	Pacemaker	73-year-old female	Not stated	Replacement of pacemaker

*ICD* Implantable cardioverter-defibrillator, *ADHD* attention deficit hyperactivity disorder, *DBS* deep brain stimulator, *IPG* internal pulse generator

Reported measures to reduce the incidence of IPG displacement and Twiddler’s syndrome include suture sleeves [27, 30]. Others have used prolene mesh, non-absorbable sutures, anchoring the IPG to the fascia, placing the IPG in a submuscular plane and limiting the pocket size to prevent further occurrences of Twiddler’s syndrome [3, 17, 21, 31, 34]. We suggest that the relocation of the IPG to above the iliac crest allows the IPG to be anchored to the lumbar fascia. Twiddler’s syndrome maybe less likely to occur with an IPG in this position as it is less accessible and visible to the patient. This approach has the added advantage of good access to the spine lead and IPG site simultaneously in the prone position. Another measure that may help prevent Twiddler’s syndrome may be psychological intervention in those identified at risk of manipulating their IPGs [22].

## Conclusions

To the best of our knowledge, this is the first reported series of Twiddler’s syndrome with IPGs for spinal cord stimulation. There are many factors that may contribute to Twiddler’s syndrome including the patient’s weight, site of implantation and psychological disorders. Based on our case series, implanting the IPG in the lumbar region subcutaneously above the iliac crest may prevent recurrence of Twiddler’s syndrome. This approach has the added advantage of easier access to the spine with tunnelling in the prone position. Psychological screening may aid in identifying those at risk of Twiddler’s syndrome.
